# The impact of family farming on Afrotropical flower fly communities (Diptera, Syrphidae): A case study in Tanzania

**DOI:** 10.1371/journal.pone.0327126

**Published:** 2025-07-01

**Authors:** Sija Kabota, Jacqueline Bakengesa, Jenipher Tairo, Abdul Kudra, Ramadhani Majubwa, Marc De Meyer, Maulid Mwatawala, Kurt Jordaens, Massimiliano Virgilio

**Affiliations:** 1 Department of Crop Science and Horticulture, Sokoine University of Agriculture (SUA), Morogoro, Tanzania; 2 Research, Consultancy and Publication Unit, National Sugar Institute (NSI), Kidatu-Morogoro, Tanzania; 3 University of Dodoma, Biology Department, Dodoma, Tanzania; 4 Invertebrates Section and JEMU, Royal Museum for Central Africa, Tervuren, Belgium; Sunrise University, INDIA

## Abstract

To provide empirical evidence about the impact of family farming on Afrotropical flower fly communities (Diptera, Syrphidae), we established a large experimental setup in the Morogoro area (Eastern Central Tanzania) and quantified insect abundance and diversity in contrasting agricultural landscapes. Over the two years of this study, we collected 12,969 flower flies from 55 species and 3 subfamilies: Eristalinae (29 species), Microdontinae (2 species), and Syrphinae (24 species). The ten most abundant species contributed to 84.95% of specimens. Overall, we did not observe major changes in species richness or diversity between agroecological and conventional farming. In contrast, higher abundances of the two dominant species, *Toxomerus floralis* (Fabricius, 1798) and *Paragus borbonicus* Macquart, 1842 (69.49% of all specimens collected) were observed in agroecological treatments. This effect was more pronounced where the landscape features were more favourable to each of these species (i.e., in the plateau for *T. floralis* and in the mountains for *P. borbonicus*). Landscape provided a comparably much stronger effect than farming practices, and the percentage of variation explained by landscape, as a standalone factor, was approximately five times higher than for farming practices. Spatial heterogeneity and seasonality also provided a large and significant proportion of random variability. Our results stress how verifying a generally accepted paradigm of sustainable agriculture, “*agroecology promotes abundance and diversity of beneficial insects*”, might require careful consideration, as, under field conditions, the impact of sustainable farming practices on insect communities might be embedded within complex, multi-layered ecological interactions.

## Introduction

Insects of agricultural importance, e.g., bees (Hymenoptera, Apidae) and flower flies (Diptera, Syrphidae), have significant impacts on crop production and food security in Sub-Saharan Africa [1–3]. In this context, agroecological systems, being, by nature, more diverse than conventional agricultural systems, are expected to promote the abundance and diversity of beneficial insects and to increase the quantity, quality and stability of crop yields [4–6]. One central paradigm of agroecology is that promoting plant and animal biodiversity improves the performance of crucial insect ecosystem services, notably pollination and biological pest control [7,8]. For example, intercropping and crop rotation combine complementary plant species and increase landscape spatial and temporal diversity [9]. Conversely, non-sustainable land use and industrial pesticides threaten welfare of beneficial insect communities and their positive effects on crop production [3,10,11]. Regardless of the need for the agroecological transition [12–14], empirical evidence about the impact of agroecological systems on Afrotropical insect diversity virtually does not exist, and baseline and quantitative information is needed to relate insect community structure and diversity to ecosystem services and crop production. This need is even more urgent in a rapid climate change scenario, which is heavily impacts the agricultural production of Sub-saharan Africa [15].

With over 6,200 known species worldwide distributed, flower flies are significant contributors to crop pollination, visiting at least 72% of global food crops [16]. Adult flower flies feed on nectar and pollen [17], facilitate the transfer of the latter between flowers and enhance fruit set and quality [18]. The larvae of many flower fly species are predatory, playing a vital role in pest control [1,19], particularly aphids, which are common pests in cucurbit crops [20–22]. This dual functionality of flower flies as pollinators and pest controllers [23] presents a crucial resource for sustainable agriculture, potentially improving crop yields while reducing reliance on chemical pesticides. Syrphidae are known to positively affect crop production [16,23]. For example, Syrphidae flower fly visitation has been shown to increase strawberry yields by over 70% and double the proportion of marketable fruit [24]. While on oilseed rapes, flower flies promote a significant increase in both seed sets and yield [25]. Flower flies have also been proven to be effective predators of aphids in sweet peppers [26] and Cabbage crops [27].

Like other insect pollinators, flower fly populations face significant challenges due to habitat loss, pesticide exposure, and climate change [3,28,29]. The decline in pollinator populations, including flower flies, poses a substantial threat to agricultural productivity and ecosystem stability [29–31]. Inadequate pollination is often a result of reduced pollinator biodiversity [32–35], and it can lead to a decrease in fruit quality, particularly in Cucurbitaceae [18,36,37].

The biodiversity of flower flies in agricultural landscapes can be shaped by various factors, including farming practices [38,39], altitude [19,40], and seasonal variations [41–43]. Recent studies indicate how agroecological practices promote flower fly diversity compared to conventional farming [3,44–47]. For example, research by Lichtenberg et al. [48] and Steffan-Dewenter [49] demonstrated that agroecological practices create a more hospitable environment for pollinators and natural enemies by fostering floral diversity and habitat complexity. These practices emphasize biodiversity, ecological balance, and sustainable resource management, fostering a more hospitable environment for flower flies by providing a continuous supply of floral resources and reduced exposure to harmful agrochemicals [29,30,47,50]. In contrast, conventional farming, characterized by monocultures and high chemical inputs, can lead to habitat loss and reduced biodiversity, with a negative impact on flower fly populations [29,51,52]. Despite the growing body of research on flower flies in agroecosystems, there remains a significant gap in our understanding of flower fly communities in Africa. A study by Rweyemamu et al. [47] in Morogoro, Tanzania, provided preliminary insights into how different agricultural practices influence the foraging behaviour of flower fly pollinators in cucurbit crops. Additionally, Kabota et al. [53] examined how variations in altitude and crop species affect flower fly diversity and floral visitation patterns in cucurbit crops. However, a comprehensive characterization of flower fly communities in Afrotropical farming systems is still lacking. Therefore, addressing this gap is crucial for developing sustainable agricultural practices that support pollinator conservation to enhance pollination services in African agroecosystems.

In Tanzania, more than 65% of the population is involved in agriculture activities. Approximately 19 million people farm 3.7 million smallholdings (between 0.9 to 3 ha) and contribute to more than 75% of agricultural production [12,54,55]. In this context, the agroecological transition is highly desirable to foster sustainable food production and social equity while ensuring environmental conservation [14,55–57], and is currently supported by national strategies and initiatives [58–60]. According to Bakengesa et al. [61], smallholder Tanzanian farmers can be roughly divided into two partially overlapping categories: those with such a low income that they cannot afford chemical pesticides and fertilizers and those who have access to and actively rely on synthetic pesticides [61]. This distinction influences farming practices and, consequently, agroecosystem dynamics.

In this study, we focus on the family farming of cucurbit crops as a typical agroecosystem in Tanzania, which has already been the target of socioeconomic [62] and entomological research [47,63,64]. Given the critical role of pollinators in agricultural productivity, understanding how different farming practices influence flower fly (Syrphidae) communities is essential. We hypothesize that agroecological farming practices support greater flower fly diversity and abundance than conventional farming due to the more stable availability of key resources such as food, shelter, and larval habitats across seasons and agroecological zones.

To test this hypothesis, here, we provide a first characterization of the Syrphidae communities and evaluate differences between agroecological and conventional farming, under the prediction that *“flower fly species richness and abundance increase in complex agricultural landscapes incorporating features that provide a temporarily stable supply of resources such as food, shelter and larval habitat”* [16]. With this objective, we established a large experimental setup in the Morogoro area (Eastern Central Tanzania) and quantified flower fly abundance and diversity in contrasting agricultural landscapes and over multiple cropping seasons.

## Materials and methods

### Study area and experimental design

This research was carried out between April 2022 and November 2023 in a large experimental setup established in the Morogoro region (Central-Eastern Tanzania). This area includes two main landscapes: the “plateau”, with an altitude between 300 and 600 m, annual rainfall between 700 and 1200 mm, and an average temperature of 29ºC, and the “mountainous landscape”, with an altitude between 600 and 900 m, annual rainfall of 800–2500 mm, and an average temperature of 24ºC [65]. In an area of approximately 80 km^2^, we selected 20 small farms (10 in each landscape) where either agroecological or conventional land management was implemented for at least four years. The distance between farms was approximately 1 km.

**Fig 1:**
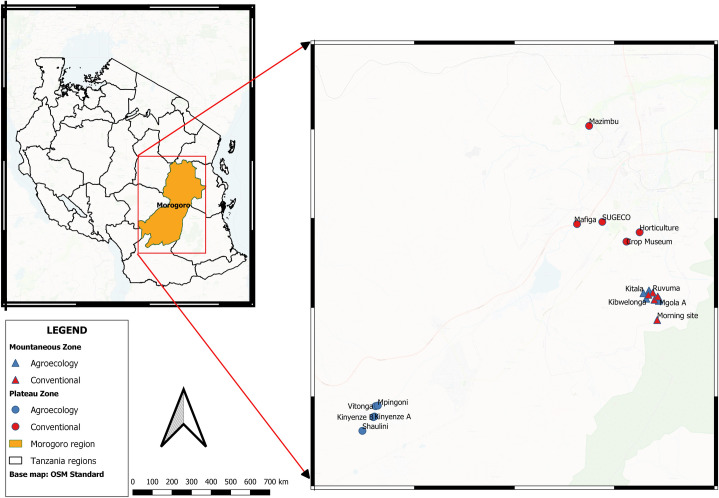
Map of the experimental sites in Morogoro, Tanzania (see Acknowledgements for map copyright notice).

In each farm, we established an experimental plot of 45 m x 45 m (2,025 m^2^), which was further subdivided into three subplots of 15 x 45 m where either cucumber (*Cucumis sativus* L.) or watermelon (*Citrullus lanatus* (Thunb.) Matsum. & Nakai), or squash (*Cucurbita moschata* D.) were planted (at a spacing of 50 cm x 60 cm, 1.5 m x 1.5 m, and 1.5 m x 1.5 m, respectively). The distance between zones was more than 8 km. Within each zone, five plots were assigned agroecological and 5 to conventional crop management (Fig 1).

Agroecological cucurbit management included manual weeding, animal manuring, mulching, intercropping, border crops and organic control of insect pests and diseases, while conventional management relied on industrial pesticides and fertilizers. The detailed protocols for these two treatments are detailed in S1Table. The experimental plots were maintained for two years, and the crops were planted, cultivated and harvested during four cropping seasons in 2022 and 2023 (April-June 2022, September-November 2022, April-June 2023, September-November 2023). During each season, flower flies were sampled once per week for eight consecutive weeks. Each weekly sampling included three sweep netting on established transects carried out for 15 minutes in each subplot and three yellow pan traps deployed in each subplot and emptied after 48 hours. The flower flies were collected, preserved in 100% ethanol, and morphologically identified by Sija Kabota and Kurt Jordaens using the available identification keys, including the Manual of Afrotropical Diptera Volume 2 [66]. Following established procedures, reference specimens of the collected species were deposited at the Sokoine University of Agriculture (SUA) and the Royal Museum for Central Africa (RMCA) in Belgium.

### Ethics statement

This study protocol was approved by the Ethics Review Board of the College of Agriculture, Sokoine University of Agriculture, Tanzania (Approval Number: PCS/D/2021/0024/02). Written informed consent was waived by the Ethics Review Board, as the study was conducted entirely within the land owned by Sokoine University of Agriculture and partner (Sustainable Agriculture Tanzania (SAT)). As the Nagoya Protocol on Access and Benefit-sharing (ABS) is *de facto* not implemented in Tanzania, the intellectual and physical property of samples collected in this study is regulated by Mutually Agreed Terms (MATs) on the use of genetic resources established between SUA and RMCA. This document, which is inspired and fully adheres to the principles of the Nagoya protocol, is provided as a supplementary S1File.

### Inclusivity in global research

Additional information regarding the ethical, cultural, and scientific considerations specific to inclusivity in global research is included in the Supporting Information (S2 File).

### Data analysis

To maximize sampling representativeness and increase the power of the tests, we adopted a total evidence approach, which consisted of pooling data collected from sweep netting and pan trapping for each weekly sampling. This produced a dataset of 11,520 samples collected across two years. Alpha diversity (α) was estimated by Species richness (S) and evenness (J) as well as by the Shannon-Weiner index (H). The effects of management practices (MP: agroecological vs conventional), landscape (ZO: plateau vs mountainous), season (SE: S1, S2,S3,S4), and plot (PL) on species abundances and diversity were tested via Analysis of Variance (ANOVA) as implemented by General Analysis of Variance Design (GAD) [67]. Homoscedasticity was preliminarily verified via Cochran’s C test, and the data were transformed when required [68]. The Student- Newman-Keuls (SNK) test was used for *a posteriori* comparisons of means [69]. Multivariate location and dispersion effects on β diversity were tested via Permutational Multivariate Analysis of Variance (PERMANOVA) [70] based on Euclidean distances and the same multifactorial design considered for univariate data. To reduce the weight of dominant taxa and better detect possible changes in the abundance of taxa, we fourth-root transformed the data as recommended by Clarke [71]. PERMANOVA was based on 99,999 iterations of residuals under a reduced model as implemented in Primer-e 7.0.21 [72]. *A posteriori* pairwise comparisons of factor levels were implemented via permutational t-statistics [70]. Patterns of β diversity were visualized using unconstrained (non-metric Multidimensional Scaling) and constrained ordinations (Analysis of Principal Coordinates) [73,74].

## Results

### Distribution of the dominant species

**Fig 2:**
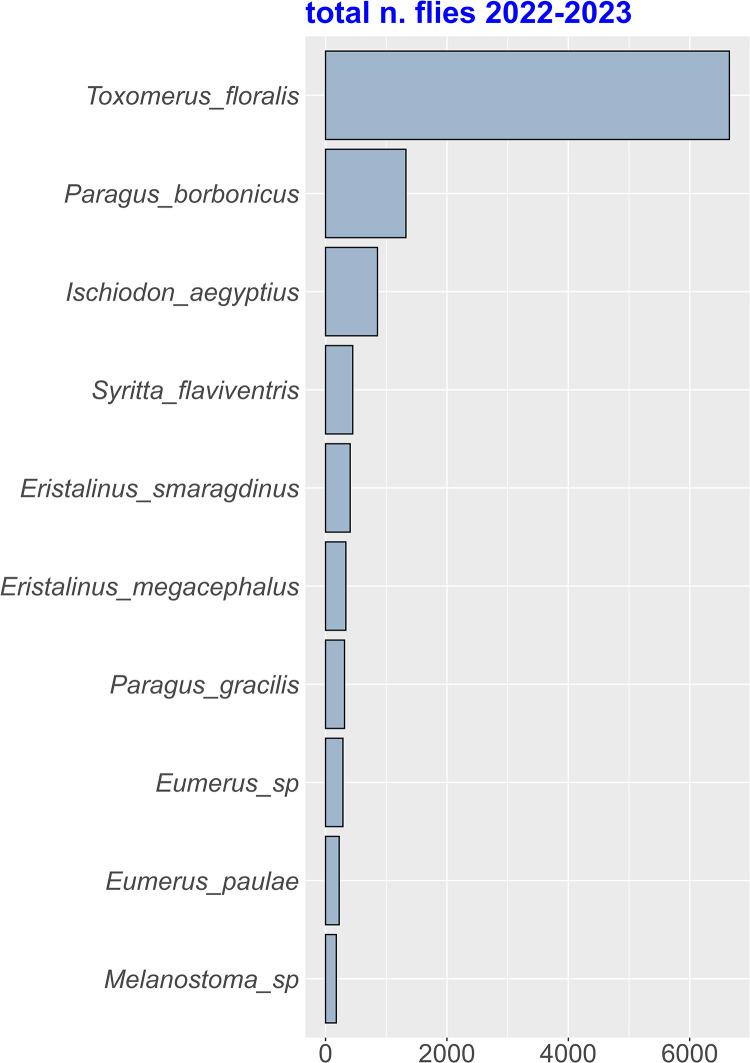
Total number of specimens for the ten most abundant flower fly species in the experimental setup.

Over the two years of this study, we collected 12,969 flower flies from 55 species and 3 subfamilies, Eristalinae (29 species), Microdontinae (2 species), and Syrphinae (24 species) (S2 Table). Ten most abundant species (Fig. 2) contributed to 84.95% of total abundances. Among them, *Toxomerus floralis, Paragus borbonicus* and *Ischiodon aegyptius* (Wiedemann, 1830) contributed to 51.3%, 10.2% and 6.6% of total abundances and represented 68.1% of all specimens collected. The patterns of distribution of flower flies were significantly affected by the random factors, season and plot (Table 1).

**Table 1:** Analysis of Variance (ANOVA) of total fly abundance and abundances of the dominant species, *T. floralis, P. borbonicus, I. aegyptius*. Test for the effects of Management Practice (MP: agroecological vs conventional), Landscape (ZO: plateau vs mountainous), Season (SE: S1, S2, S3, S4), and Plot (PL: 20 plots). The results of the *post hoc* tests are indicated for the significant interactions with the fixed factors (in blue: MPxZO, MPxSE, ZOxSE and MPxZOxSE). ns: not significant; *: P < 0.05; **: P < 0.001; ***: P < 0.0001.

		total n. of flies				*Toxomerus floralis*				*Paragus borbonicus*				*Ischiodon aegyptius*			
Source of Variations		ANOVA, square root transf. data, C = 0.023, n.s.				ANOVA, untransformed data, C = 0.029, n.s				ANOVA, fourth root transf. data, C = 0.019, n.s.				ANOVA, fourth root transf. data, C = 0.024, n.s.			
	df	MS	F	P		MS	F	P		MS	F	P		MS	F	P	
Management Practice (MP)	1	0.604	0.069	0.797	n.s.	1.087	5.900	0.027	*	0.841	8.488	0.010	*	1.059	3.477	0.081	ns
Landscape (ZO)	1	128.710	14.616	0.001	**	18.132	98.424	0.000	***	0.857	8.654	0.010	*	1.459	4.790	0.044	*
Season (SE)	3	121.336	21.246	0.000	***	8.728	86.752	0.000	***	0.726	8.465	0.000	***	2.062	19.728	0.000	***
MP × ZO	1	11.133	1.264	0.277	n.s.	1.965	10.665	0.004	**	0.777	7.848	0.013	*	0.783	2.571	0.128	ns
MP × SE	3	27.368	4.792	0.005	**	0.241	2.397	0.080	ns	0.122	1.427	0.247	ns	0.694	6.643	0.001	**
ZO × SE	3	108.347	18.972	0.000	***	5.828	57.925	0.000	***	0.437	5.096	0.004	**	1.953	18.681	0.000	***
Plot: PL (ZO x MP)	16	8.806	2.595	0.001	***	0.184	3.265	0.000	***	0.099	1.061	0.389	ns	0.305	4.115	0.000	***
MP x ZO x SE	3	13.699	2.399	0.079	n.s.	0.444	4.414	0.008	**	0.020	0.230	0.875	ns	0.196	1.878	0.146	ns
SE x PL (Zox MP)	48	5.711	1.683	0.003	**	0.101	1.783	0.000	***	0.086	0.918	0.633	ns	0.105	1.412	0.035	*
Residual	1360					0.056				0.093				0.074			
*Post hoc tests*																	
MP						agroecological > conventional				agroecological > conventional							
ZO		mountainous < plateau				mountainous < plateau				mountainous > plateau				mountainous < plateau			
MP x ZO						mountainous	agroecological = conventional			mountainous	agroecological > conventional						
						plateau	agroecological > conventional			plateau	agroecological = conventional						
MP x SE		S1:	agroecological = conventional											S1:	agroecological = conventional		
		S2:	agroecological < conventional											S2:	agroecological < conventional		
		S3:	agroecological > conventional											S3:	agroecological = conventional		
		S4:	agroecological = conventional											S4:	agroecological = conventional		
ZO x SE		S1:	mountainous < plateau			S1:	mountainous < plateau			S1:	mountainous > plateau			S1:	mountainous > plateau		
		S2:	mountainous = plateau			S2:	mountainous = plateau			S2:	mountainous = plateau			S2:	mountainous = plateau		
		S3:	mountainous < plateau			S3:	mountainous < plateau			S3:	mountainous = plateau			S3:	mountainous = plateau		
		S4:	mountainous > plateau			S4:	mountainous = plateau			S4:	mountainous > plateau			S4:	mountainous < plateau		
MP x ZO x SE						mountainous	S1:	agroecological < conventional									
						mountainous	S2:	agroecological = conventional									
						mountainous	S3:	agroecological = conventional									
						mountainous	S4:	agroecological = conventional									
						plateau	S1:	agroecological > conventional									
						plateau	S2:	agroecological = conventional									
						plateau	S3:	agroecological > conventional									
						plateau	S4:	agroecological = conventional									

**Fig 3:**
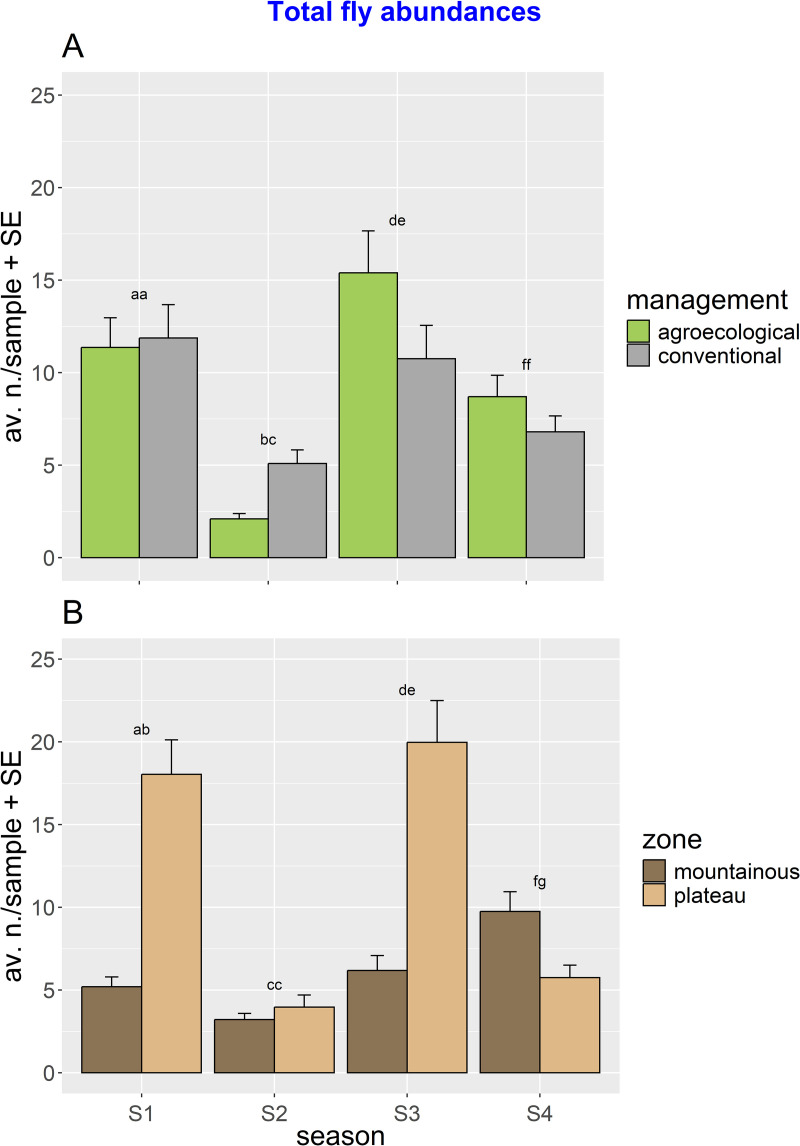
Total flower fly abundances. The bar plots illustrate the significant interactions: (A) Management x season (MPxSE) and (B) zone x season (ZOxSE). Significance letters for the pairwise tests are indicated (see Table 1).

ANOVA showed how flower fly abundances were significantly higher in the plateau (where 66.2% of specimens were collected), compared to the mountainous landscape (33.8% of specimens). The significant interactions of MPxSE and ZOxSE showed that the spatiotemporal distribution of flower flies was strongly affected by seasonality (Fig. 3). The *post hoc* tests showed variable seasonal patterns both across managements and zones (i.e., with higher flower fly abundances in conventional management at season 2 and in agroecological management at seasons 3 and 4, and higher flower fly abundances in the plateau at seasons 1 and 3 and in the mountainous landscape at season 4).

**Fig 4:**
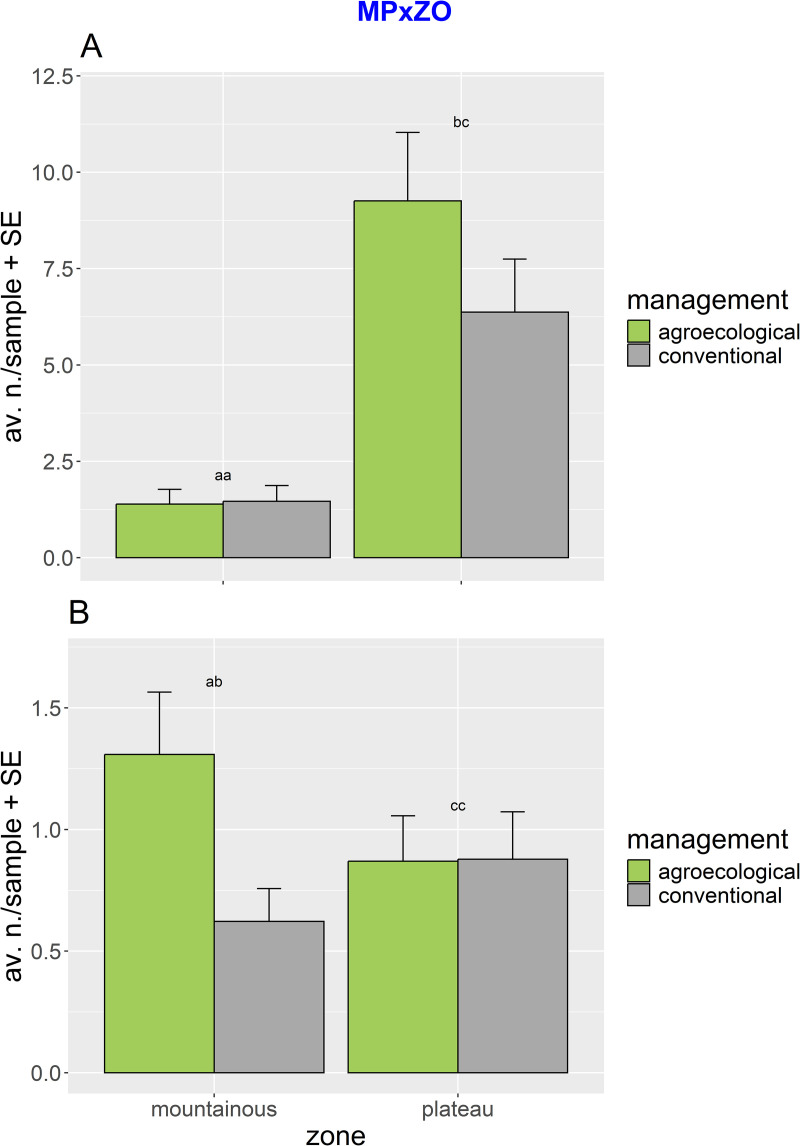
Abundances of *T. floralis* and *P. borbonicus.* The bar plots illustrate the significant interaction of management x zone (MPxZO) on abundance: (A) for *T. floralis* and (B) for *P. borbonicus*. Significance letters for the pairwise tests are indicated (see Table 1).

The overall abundance of *T. floralis* was significantly higher in agroecological farming. This species, which was also more abundant in the plateau (Table 1) showed significant seasonal changes. The *post hoc* tests for the significant interaction MPxZOxSE show variable seasonal patterns both across management and zones (i.e., with higher abundances of *T. floralis* in the mountainous landscape from agroecological management at season 1 and 3 and higher abundances in the plateau from conventional management at season 1) (S1Fig). Regardless of the random spatiotemporal variation, the significant interaction of MPxZO and the posthoc tests revealed how, in the plateau, the overall abundance of *T. floralis* was significantly higher in agroecological compared to conventional management, while no significant differences were observed in the mountainous landscape (Fig. 4). Also, the second most abundant species, *P. borbonicus*, was significantly more abundant in agroecological farming and showed differential distributions between zones, with higher abundances in the mountains (Table 1). ANOVA showed significant effects related to the interaction of MPxZO. The *post hoc* comparisons for the significant interaction MPxZO revealed that in the mountainous landscape, the abundance of *P. borbonicus* was significantly higher in agroecological compared to conventional management, while no significant differences were observed in the plateau (Fig. 4). Also, this species was affected by significant seasonal variation, with higher abundances in the mountainous landscape detected during 3 out of the 4 seasonal samplings, and the exception of season 2 when this pattern was reversed. *Ischiodon aegyptius* showed comparable distributions between agroecological and conventional farming and higher abundances in the plateau. Also, this species showed seasonal variability, which was reflected by the significant interactions of ZOxSE and MPxSE. The *post hoc* tests revealed how the abundance of this species during season 1 was significantly higher in the mountainous landscape compared to the plateau, while in season 4, this pattern was reversed, and no significant differences were observed in season 2 and season 3. Additionally, *I. aegyptius* showed distributional differences between agroecological and conventional management during one of the four sampling seasons (S2Fig).

### Patterns of α and β diversity

**Fig 5:**
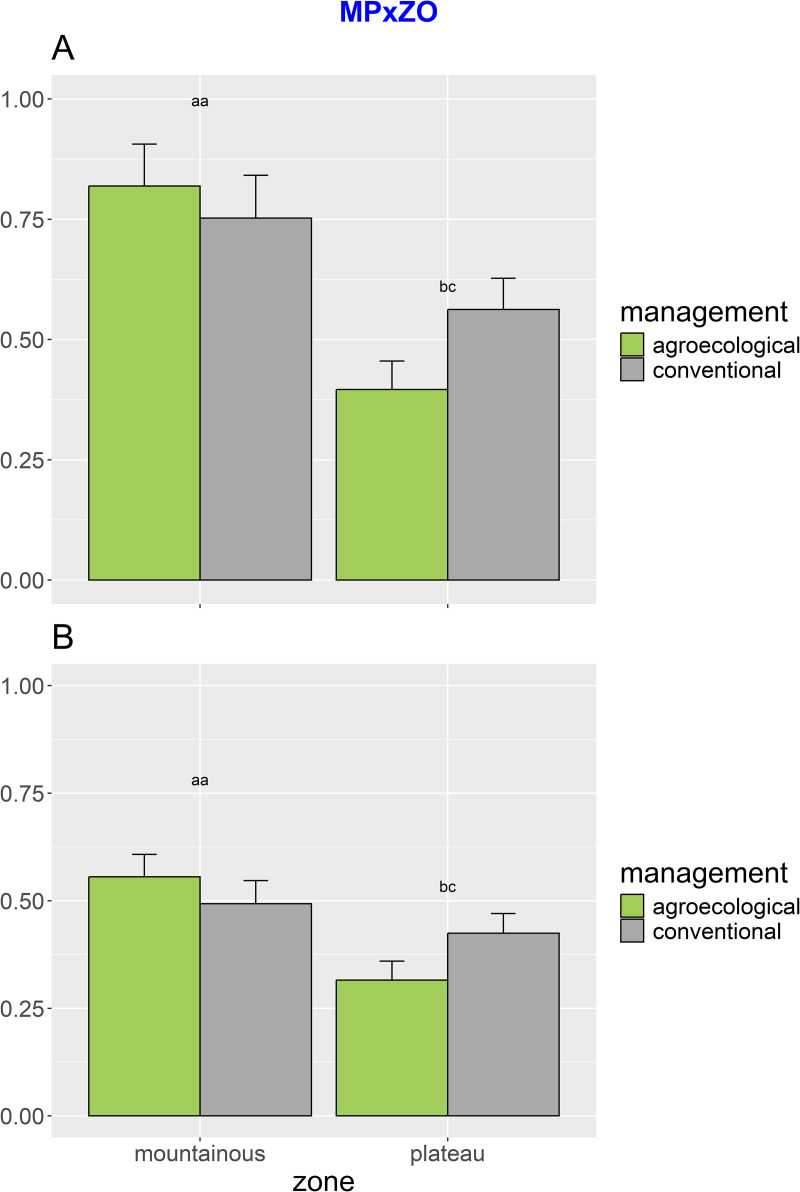
Shannon Diversity and evenness. The bar plots illustrate the significant interactions of management x zone (MPxZO) on (A) Shannon diversity and (B) species Evenness. Significance letters for the pairwise tests are indicated (see Table 2).

Also all the α diversity estimators were significantly affected by the random factors, season and plot (Table 2). ANOVA on species richness showed significantly higher species richness in the mountainous landscape and significant interactions of MPxSE and ZOxSE (S3 Fig). The *post hoc* tests revealed variable seasonal patterns both across managements and zones, i.e., with higher species richness in conventional management during one of the sampling seasons (season 2) and higher species richness in the mountainous landscape during seasons 2 and 4 (Table 2). Shannon Diversity was significantly higher in the mountainous landscape and affected by the interactions of MPxZO (Fig. 5) and MPxSE (S4Fig). The *post hoc* tests showed that, in the plateau, Shannon Diversity was higher in conventional compared to agroecological management both in the plateau and during season 2 (Table 2). Also Evenness was significantly higher in the mountainous landscape. We observed significant effects of the interactions of MPxZO (Fig 5) and MPxZOxSE (S5Fig). The *post hoc* tests revealed higher evenness in conventional farming in the plateau but not in the mountainous landscape as well as variable seasonal patterns with higher evenness in agroecological farming on the mountains at seasons 3 and in conventional farming in the plateau at season 2 (Table 2).

**Table 2:** Patterns of α diversity (ANOVA of Species richness, Evenness J, Shannon diversity) and β diversity (PERMANOVA of 55 flower fly species). Test for the effects of Management Practice (MP: agroecological vs conventional), Landscape (ZO: plateau vs mountainous), Season (SE: S1, S2, S3, S4), and Plot (PL: 20 plots). The results of the post hoc tests are indicated for the significant interactions with the fixed factors (in blue: MPxZO, MPxSE, and ZOxSE). ns: not significant; *: P < 0.05; **: P < 0.001; ***: P < 0.0001.

		Species Richness				Evenness J				Shannon diversity				β diversity				
		ANOVA, untransformed data, C = 0.027, n.s.				ANOVA, untransformed data, C = 0.016, n.s.				ANOVA, untransformed data, C = 0.022, n.s				PERMANOVA, 4th root transf data, Euclidean distance				
Source of Variations	df	MS	F	P		MS	F	P		MS	F	P		MS	F	P		% variation
**Management Practice (MP)**	1	2.178	0.138	0.715	n.s.	0.192	0.470	0.503	n.s.	0.896	1.088	0.312	n.s.	21.625	1.226	0.175	n.s.	1.9%
**Landscape (ZO)**	1	189.225	11.979	0.003	**	8.568	20.955	0.000	***	33.827	41.082	0.000	***	193.460	2.993	0.007	**	9.8%
**Season (SE)**	3	114.917	13.730	0.000	***	0.182	1.036	0.385	n.s.	3.330	8.524	0.000	***	91.560	16.753	0.000	***	11.2%
**MP × ZO**	1	55.225	3.496	0.080	n.s.	2.643	6.463	0.022	*	4.887	5.935	0.027	*	28.884	1.829	0.010	*	4.7%
**MP × SE**	3	31.991	3.822	0.016	*	0.460	2.624	0.061	n.s.	1.342	3.434	0.024	*	11.276	2.063	0.002	**	4.1%
**ZO × SE**	3	55.575	6.640	0.001	***	0.452	2.576	0.065	n.s.	0.288	0.738	0.535	n.s.	55.646	10.182	0.000	***	12.1%
**Plot: PL (ZO x MP)**	16	15.797	2.391	0.002	**	0.409	2.404	0.001	**	0.823	2.013	0.010	*	10.827	1.981	0.000	***	6.2%
**MP x ZO x SE**	3	9.782	1.169	0.331	n.s.	0.699	3.986	0.013	*	0.967	2.477	0.073	n.s.	7.952	1.455	0.057	n.s.	3.8%
**SE x PL (Zox MP)**	48	8.370	1.267	0.106	n.s.	0.175	1.031	0.415	n.s.	0.391	0.955	0.562	n.s.	5.465	2.055	0.000	***	9.0%
**Residual**	1360	6.606				0.170				0.409				2.660				37.2%
***Post hoc tests***																		
**MP**																		
**ZO**		mountainous > plateau				mountainous > plateau				mountainous > plateau				mountainous ≠ plateau				
																		
**MP x ZO**						mountainous	agroecological = conventional			mountainous	agroecological = conventional			mountainous	agroecological = conventional			
						plateau	agroecological < conventional			plateau	agroecological < conventional			plateau	agroecological = conventional			
**MP x SE**		S1:	agroecological = conventional							S1:	agroecological = conventional			S1:	agroecological ≠ conventional			
		S2:	agroecological < conventional							S2:	agroecological < conventional			S2:	agroecological + conventional			
		S3:	agroecological = conventional							S3:	agroecological = conventional			S3:	agroecological ≠ conventional			
		S4:	agroecological = conventional							S4:	agroecological = conventional			S4:	agroecological = conventional			
**ZO x SE**		S1:	mountainous = plateau											S1:	mountainous ≠ plateau			
		S2:	mountainous > plateau											S2:	mountainous ≠ plateau			
		S3:	mountainous = plateau											S3:	mountainous ≠ plateau			
		S4:	mountainous > plateau											S4:	mountainous ≠ plateau			
**MP x ZO x SE**						mountainous	S1:	agroecological = conventional										
						mountainous	S2:	agroecological = conventional										
						mountainous	S3:	agroecological > conventional										
						mountainous	S4:	agroecological = conventional										
						plateau	S1:	agroecological = conventional										
						plateau	S2:	agroecological < conventional										
						plateau	S3:	agroecological = conventional										
						plateau	S4:	agroecological = conventional										

**Fig 6:**
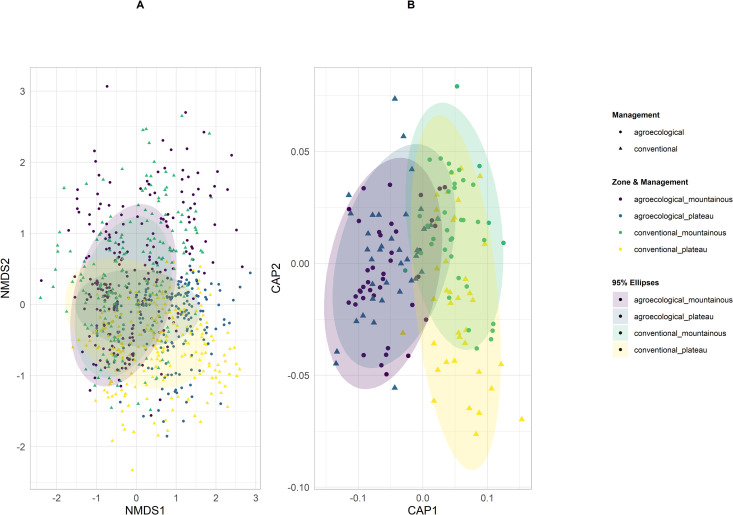
Patterns of β diversity. Interaction of the effects of management and zone (MPxZO, see Table 2). (A) Unconstrained and constrained ordinations (non-metric Multidimensional Scaling, and (B) Analysis of Principal Coordinates) of the abundances of 55 flower fly taxa in different zones (mountainous, plateau) and land management (agroecological, conventional). Results are shown for the whole dataset (1,440 samples) and for pooled replicates across sites (160 samples). Ellipses including 95% of samples are indicated.

PERMANOVA on beta diversity (Table 2) showed significant differences between landscapes and a significant interaction between Management practice and landscape (the latter providing 10.3% contribution to variation, the former 2.3%). Yet, the *post hoc* comparisons did not detect significant differences between agroecological and conventional farming either in the plateau or the mountains (MPxZO (Fig. 6)). The analyses also detected significant spatio-temporal variability associated with the random factors, season (contributing to 10.7% of variation), and plot (6.3%). The post hoc tests show how the effects of agroecological and conventional management are significantly affected by seasonality, as these could be observed in seasons 1, 2 and 3 but not in season 4 (Table 2).

## Discussion

In this work, we compared contrasting agricultural landscapes and described the effects of sustainable farming practices on the flower fly communities of an Afrotropical agroecosystem. Agroecological farming had a detectable impact on flower fly distributions, yet its effects interacted at multiple levels, generating complex spatiotemporal patterns. First, we did not observe any detectable effects of agroecological farming on flower fly species richness or overall diversity (but see below). This finding contrasts with the general paradigm linking sustainable agricultural practices to enhanced (insect) diversity [38,39,75], as well as with trends reported in other studies where agroecological systems have been shown to increase the richness and diversity of beneficial insects [76–78]. Regardless of that, agroecology had a positive impact on the abundance of the two dominant flower fly species, *T. floralis* and *P. borbonicus*, in the Morogoro area. Remarkably, these effects were only apparent under specific landscape conditions. *T. floralis* responded positively in the plateau, and *P. borbonicus* in the mountains. In other words, an agroecological effect on the dominant flower flies was only observed where landscape features were favorable to their occurrence and supported significantly higher abundances. These patterns further suggest that both species are highly responsive to habitat features that align with their specific ecological requirements [53,79,80]. Unfortunately, our understanding of the ecology of most Afrotropical flower flies remains limited [53]. Much of the available data are extrapolated from studies on Palearctic or Neotropical congeners [79,81–84]. The high abundance of *T. floralis* in plateau areas can be linked to its close association with ruderal plant species and cucurbit crops, which are common in cultivated areas in the Afrotropical region [53,85]. The larvae of *T. floralis* are known pollen-feeders, primarily utilizing *Cyperus rotundus* L. (Cyperaceae) and *Mitrocarpus hirtus* (L.) DC (Rubiaceae) as host plants [85], while adults forage on cucurbit flowers for nectar and pollen [47,53]. Both *C. rotundus* and *M. hirtus* are typical of disturbed habitats and are widespread in agricultural areas, including in the cucurbit farms in the plateau zones of the Morogoro region ( [47,53,61], SK, personal observation). Therefore, the abundance of larval host plants and the consistent presence of cucurbit crops alongside the species’ adaptability to disturbed agroecosystems likely explain the higher population of *T. floralis* observed in plateau landscapes. In contrast, *P. borbonicus* was significantly more abundant in the mountainous sites, indicating a preference for cooler climates and structurally diverse habitats. Although specific ecology details of *P. borbonicus* are still limited in the Afrotropic region [53,86–89], the available insights suggest that their larvae are aphidophagous [86,87,90], and is commonly associated with aphid colonies on herbaceous plants in semi-natural or less intensively managed environments [87–89,91]. Mountain landscapes typically support a higher diversity and abundance of aphid populations, especially in ecologically managed or less disturbed fields [84], which may explain the higher abundance of species in these areas. Additionally, like other flower fly species, *Paragus* species are known for their adaptability to high-elevation conditions [19,92,93], with records of some species occurring well above 1,500 meters [19,92,94].

Having established the main context for the agroecological effect on the dominant flower flies, it is also important to consider the relative contributions of other factors. The overall abundance of flower flies during the two years of this study was higher in the plateau, where approximately 2/3 of specimens were collected. In this context, the percentage of variation explained by landscape, as a standalone factor, was approximately five times higher than the variation explained by farming practices and shows how landscape provides a much stronger effect compared to farming practices. In other terms, where the community was sampled (in the mountains or the plateau) had comparably more weight than how the land was cultivated (conventionally or agroecologically).

Changes in the abundance of the dominant species also affected the community diversity patterns. Dainese et al. [29], in a large metadata analysis, observed that the most abundant pollinating species generally contribute more strongly to pollination service, and that, for this reason, agroecological systems supported by an effective pollination service tend to have lower evenness. This seems to be in line with the patterns observed in our experimental setup, where the higher dominance of the most abundant species, *T. floralis,* in agroecological farming (in the plateau but not in the mountains) corresponded to significantly higher evenness in conventional farming (in the plateau but not in the mountains). Based on these patterns, we speculate that the agroecological farms of the Morogoro area might benefit from more efficient flower fly pollination than conventional farms due to the contribution provided by dominant flower fly species.

Although farming practices alone did not significantly affect Shannon diversity, their interaction with landscape led to significantly higher diversity in conventional farms on the plateau. This unexpected result may be linked to the greater flight capacity of flower flies compared to other insect groups [95]. For example, cucurbit-feeding Tephritidae are confined to crops during their larval stages and tend to have shorter adult dispersal ranges [96]. In contrast, Syrphidae are likely better able to move across the patchy landscape of small orchards, allowing them to recolonize treated areas more effectively and partially offset local mortality caused by insecticide applications. This interpretation gains some tentative support from observations that flower fly larvae under field conditions appear to be relatively resistant to the effects of neonicotinoids [28]. Therefore, while localized insecticide use in conventional family-farming might be effective against a range of agricultural insect pests [10,97], it likely has a comparably less impact on Syrphidae diversity. Since landscape appears to play a much larger role than farming practices, we hypothesize that the higher diversity observed in conventional farming on the plateau is likely due to the availability of heterogeneously distributed areas suitable for the establishment of flower flies. This heterogeneity in habitat distribution may have mitigated or masked the detection of localized effects due to insecticide spraying.

Interestingly, the patterns observed for flower flies are consistent with what was observed in a previous study [64] conducted in the very same experimental setup and comparing the gut microbial communities of the melon fly, *Zeugodacus cucurbitae* (Diptera, Tephritidae). Also in this case, an “agroecological effect” with an impact on the gut microbial diversity of the melon fly could only be observed in one of the two landscapes (on the mountains but not in the plateau). Also in *Z. cucurbitae*, farming practices and landscape produced an interactive effect producing complex patterns of (microbial) diversity. Spatial heterogeneity (differences across the experimental plots) and seasonality (differences across cropping seasons) also provided a large portion of random variability, adding complexity to the flower fly distributional patterns and possibly masking part of the effects promoted by agroecological farming. This further highlights the importance of multiannual studies for detecting the interactive effects of agricultural practices on insects of agricultural importance, particularly in very dynamic scenarios such as those promoted by climate change. In this respect, the increase in climate variability, including frequency and intensity of extreme events and intra- and inter-seasonal changes, is having a considerable impact on Tanzanian agriculture and is expected to further increase in the forthcoming years [98,99].

Our results stress how verifying a generally accepted paradigm of sustainable agriculture, “agroecology promotes abundance and diversity of beneficial insects,” might require careful consideration, as, under field conditions, the impact of sustainable farming on insect communities could be embedded within complex, multi-layered ecological interactions. In this context, the relative contribution of pollinators’ richness, evenness, and total and relative abundances to key agroecosystem services [29] would definitely benefit from large-scale and long-term evidence from field studies.

## Supporting information

S1 File Mutually Agreed Terms (MATs) on the use of genetic resources established between SUA and RMCA. (PDF)

S2 File PLOS ONE Global Research Inclusivity Questionnaire. (DOCX)

S1Table Detailed protocols for farming practices used to manage cucurbit crops during the study. (DOCX)

S2 Table List of hoverfly species and their subfamilies collected during the entire study period in Morogoro from 2022 to 2023. (XLSX)

S3 Table Metadata: Research raw data used for this study. (XLSX)

S4 Table Minimal data set: Data used to reach the conclusions drawn in the manuscript in its entirety. (CSV)

S1 Fig Abundances of *T. floralis.* The bar plots illustrate the significant interaction of management x zone x season (MPxZOxSE) across (A) the plateau and (B) the mountainous. Significance letters for the pairwise tests are indicated (see Table 1).
(TIF)

S2 Fig Abundance of *I. aegiptyius.* The bar plots illustrate the significant interactions of: (A) management x season (MPxSE) and (B) zone x season (ZOxSE). Significance letters for the pairwise tests are indicated (see Table 1).
(TIF)

S3 Fig Species Richness. The bar plots illustrate the significant interactions of: (A) management x season (MPxSE) and (B) zone x season (ZOxSE). Significance letters for the pairwise tests are indicated (see Table 2).
(TIF)

S4 Fig Shannon Diversity. The bar plots illustrate the significant interactions of management x season (MPxSE). Significance letters for the pairwise tests are indicated (see Table 2).
(TIF)

S5 Fig Evenness. The bar plots illustrate the significant interactions of management x zone x season (MPxZOxSE) across (A) the plateau and (B) the mountainous. Significance letters for the pairwise tests are indicated (see Table).
(TIF)
