# Supplementary material for: The impact of family farming on Afrotropical flower fly communities (Diptera, Syrphidae): A case study in Tanzania
Source: PLoS One. 2025 Jul 1;20(7):e0327126. doi: 10.1371/journal.pone.0327126 (PMC12212540; doi:10.1371/journal.pone.0327126)
Supplement: S2 File — (DOCX) [file pone.0327126.s002.docx]

Inclusivity in global research

PLOS’ policy on inclusivity in global research aims to improve transparency in the reporting of research performed outside of researchers’ own country or community and ensures that PLOS publications reporting global research adhere to high standards for research ethics and authorship. Authors of relevant research articles may be asked to complete the questionnaire below, which outlines ethical, cultural, and scientific considerations specific to inclusivity in global research. This questionnaire may be requested when researchers have travelled to a different country to conduct research, if research uses samples collected in another country, research with Indigenous populations or their lands, or if research is on cultural artefacts. Researchers travelling to another country solely to use laboratory equipment will not normally be required to complete the questionnaire. However, the questionnaire can be requested at the journal’s discretion for any submission – if you have been requested to complete this questionnaire by the PLOS journal you submitted to, please do so.

Please complete the questionnaire below and include this as a Supporting Information file with your manuscript. Note that if your paper is accepted for publication, this checklist will be published with your article in the supporting information files. Please ensure that you reference the checklist in the main body of your manuscript. We suggest adding a subsection ‘Inclusivity in global research’ to your Methods section and adding the following sentence: “Additional information regarding the ethical, cultural, and scientific considerations specific to inclusivity in global research is included in the Supporting Information (SX Checklist)”

The questions have been designed to be applicable to a wide range of study types, and there are subsections for both human subjects research and non-human subjects research. If any of the questions are not relevant to your research please mark them as “N/A” as appropriate.

**Ethical considerations, permits and authorship**

*This section is applicable to all research types.*

Provide details as to who granted permissions and/or consent for the study to take place in the Methods section of your manuscript. This should include the names of **all** ethics boards, governmental organizations, community leaders or other bodies that provided approval for the study. If individuals provided approval refer to these people by their role or title but do not list their name(s).

Information included in the Ethics Statement is reported at p.8, lines 174-185.

If there were any deviations from the study protocol after approval was obtained please provide details of these changes in the Methods section of your manuscript.
Did this study involve local collaborators that are residents of the country where the research was conducted or members of the community studied? If you do not have any authors from said communities, please provide an explanation for this below.

NA

As part of the Royal Museum for Central Africa’s (RMCA) ongoing efforts toward decolonisation and inclusive research practices, this study reflects a balanced collaboration between European and African researchers. The author team includes scientists based in the country where the research was conducted, with the first author and several co-authors affiliated with the Sokoine University of Agriculture (SUA) in Morogoro, Tanzania.

All authors were engaged from the early stages of the project, including the research design, to ensure that the diverse perspectives represented were integrated from the outset. Where relevant, we cited literature published by African scientists to support inclusivity and proper recognition of regional contributions.

The field experiments were fully authorized and coordinated by SUA and were conducted in collaboration with the Tanzanian non-governmental organization Sustainable Agroecology Tanzania (SAT), based in Morogoro. The work also involved the active participation of farmers in the Morogoro region. Field assistance was provided by SUA students temporarily employed in Morogoro for this purpose.

The last author of this paper is a researcher at the RMCA and contributed to the study in the context of the institution’s broader commitment to equitable international research partnerships.

Everyone listed as an author should meet PLOS’ criteria for authorship and all individuals who meet these criteria should be included in the author byline, rather than the acknowledgements. For further information please see the journal’s Authorship Policy.

**Human subjects research (e.g. health research, medical research, cross-cultural psychology)**

Did you obtain written informed consent from a representative of the local community or region before the research took place? How did you establish who speaks for the community? Details of written informed consent obtained from study participants should be reported separately in the Methods section of your manuscript.

NA

How did members of the local community provide input on the aims of the research investigation, its methodology, and its anticipated outcome(s)?

NA

When engaging with the local community, how did you ensure that the informed consent documents and other materials could be understood by local stakeholders?

NA

Will the findings of the research be made available in an understandable format to stakeholders in the community where the study was conducted (e.g. via a presentation, summary report, copies of publications, etc.)? Please provide details of how this will be achieved.

This research was conducted as part of a broader project funded by the Belgian Science Policy (BELSPO), which aims to support sustainable agroecological development through collaborative international research. An open access project report, summarizing the main findings and their implications, will be published in 2025 to ensure the wide dissemination of results.

Importantly, the findings of this study will also be made available in accessible formats to stakeholders in the community where the research was carried out. This will include presentations and knowledge-sharing sessions at local institutions such as the Sokoine University of Agriculture (SUA), as well as through ongoing collaboration with partner organizations like Sustainable Agriculture Tanzania (SAT) and the Royal Museum for Central Africa (RMCA).

In addition, the results will be presented at relevant national and regional conferences to engage a broader audience of practitioners and decision-makers. All publications resulting from the project will be made freely available through open access platforms, and printed copies of the articles and summary materials will be distributed to key stakeholders and institutions involved in or impacted by the research.

**Non-human subjects research using specimens/ animals collected as part of the study, or those housed in archival collections. Examples include archaeology, paleontology, botany and zoology.**

Did the permission you obtained from a local authority to perform the study include an agreement on access to outputs and benefit sharing? This may include procedures to enable fair distribution of the benefits and resources arising from the research performed. Please include any details of Prior Informed Consent and Benefit Sharing Agreements obtained. These may be required by field-specific regulations, for example the Convention on Biological Diversity (CBD) and the associated Nagoya Protocol.

As part of the Ethics Statement included in the manuscript (p. 8, lines 174–185), we clarify that:

“As the Nagoya Protocol on Access and Benefit-sharing (ABS) is not formally implemented in Tanzania, the intellectual and physical ownership of samples collected in this study is governed by Mutually Agreed Terms (MATs) on the use of genetic resources, established between the Sokoine University of Agriculture (SUA) and the Royal Museum for Central Africa (RMCA). This agreement, which is inspired by and fully aligned with the principles of the Nagoya Protocol, is provided as Supplementary Material (S1File).”

We have included the full text of this agreement as Supplementary File S1 to ensure full transparency regarding access and benefit-sharing arrangements.

If the material used in your study was imported, please A) provide the year it was imported and B) indicate whether permits were obtained to import/export the materials used, C) provide details of any permits obtained. If this information is not available, please indicate this.

A small proortion of the samples collected during this research was transferred to the Royal Museum for Central Africa (RMCA) to confirm their morphological identification. The majority of the material was, or is in the process of being, returned to the Sokoine University of Agriculture (SUA). In accordance with the agreements established between SUA and RMCA (see MATs), small subsets of voucher specimens are currently held at RMCA on loan. The intellectual and physical ownership of these specimens remains with SUA.

If you used archival specimens, please state how the material used in your study was acquired by the institute it is held in and provide details of any permits obtained for the original excavations/ sample collection. If this information is not available, please indicate this.

NA

How was the potential cultural significance of the materials collected in your study to local communities considered in your research design? Were Indigenous peoples and/or local researchers and institutions involved with archaeological excavations / collection of specimens? If so, please provide a description of their involvement.

The insect species collected in this study do not have known cultural significance for communities in the study area. The research was designed and implemented in close collaboration with institutions and stakeholders based in the region. Farmers contributed to the experimental treatments by applying agroecological and conventional farming protocols, following training and coordination provided by the Sokoine University of Agriculture (SUA) and Sustainable Agriculture Tanzania (SAT). Their participation was compensated in accordance with agreements established with SUA.

Specimen collection was carried out by researchers from SUA, assisted by SUA students. This approach ensured that regional expertise was meaningfully integrated into the fieldwork, with transparent roles and responsibilities throughout the research process.

If your manuscript includes photographs of human remains please indicate whether authors obtained permission from descendants or affiliated cultural communities to do so.

NA
