# Supplementary material for: The impact of family farming on Afrotropical flower fly communities (Diptera, Syrphidae): A case study in Tanzania
Source: PLoS One. 2025 Jul 1;20(7):e0327126. doi: 10.1371/journal.pone.0327126 (PMC12212540; doi:10.1371/journal.pone.0327126)
Supplement: S1Table — (DOCX) [file pone.0327126.s003.docx]

S1Table. Detailed protocols for farming practices used to manage cucurbit crops during the study.

*Agroecological package*

| Time | Activities | Pest and soil fertility management |
| --- | --- | --- |
| During field preparations | Terraces were prepared for all fields in high altitude and ridges for low altitude | 0.5kg per of cow manure was applied per hole before planting |
| After planting | A 15 cm thickness of mulching from dried straw materials was applied in all fields under agroecology. | A bio-insecticide made from 700g of neem leaves + 200g of chill + 100g garlic + two aloe vera leaves was grided together to make a 1kg mixture, followed by soaking the mixture in 10 L of water for 12 hours, followed by adding 4 spoons of cooking oil. The obtained mixture was applied twice a week. |
| After planting | Throughout crop growth stages | Bio-fungicide made from:   - 1 L of fresh milk was diluted into 10 L of water, and 100g of baking powder was added to the mixture. The mixture was then applied twice a week. - 1kg of grided pawpaw leaves mixed with 10L of water, fermented for 24 hours, sieved and applied once after two weeks |
| After planting |  | Biofertilizers made from 50kg of fresh cow dung were sacked into a 50kg sack and soaked in 200 L of water for 21 days. The obtained liquid fertilizer was applied at 250ml per week per plant. |
| Before planting |  | Bio-fence made from pigeon peas was planted at the border surrounding all fields. |
| After planting |  | Intercropping using green gram was also done on each ridge or terrace at 50cm spacing. |
| Throughout plant growth | Weeding | Manual weeding was carried out using a hand hoe to keep the field clean and free of unwanted plants. |
| Throughout plant growth | Irrigation | During the dry season, crops were irrigated three times per week using polyethylene pipes to maintain consistent soil moisture throughout their growth cycle. |

*Conventional Package*

| Time | Activities | Pest and fertilizer management |
| --- | --- | --- |
| During field preparations | Terraces were prepared in the high-altitude fields and ridges in low-altitude | No fertilizer was applied. |
| After planting |  | 102 kg/ha of NPK (N:P: K15:9:20) industrial fertilizer was applied 14 days after seed emergence |
| At the fruit set stage | Throughout crop growth stages | 102 kg/ha of CAN (15.4:26) fertilizer was applied during the flowering stage at the interval of 14 days. |
| During the crop growth stage |  | An insecticide (Organophosphate with pyrethroids) was applied at a rate of 2.5L/hectare at intervals of 14 days. |
| During the crop growth stage |  | Fungicide (Chloroforce, chlorothalonil) was also applied at a rate of 2.5L/hectare at intervals of 14 days |
| Throughout plant growth | Weeding | Manual weeding was carried out using a hand hoe to keep the field clean and free of unwanted plants. |
| Throughout plant growth | Irrigation | During the dry season, crops were irrigated three times per week using polyethylene pipes to maintain consistent soil moisture throughout their growth cycle. |
