# Supplementary figures and images for: The impact of family farming on Afrotropical flower fly communities (Diptera, Syrphidae): A case study in Tanzania
Source: PLoS One. 2025 Jul 1;20(7):e0327126. doi: 10.1371/journal.pone.0327126 (PMC12212540; doi:10.1371/journal.pone.0327126)

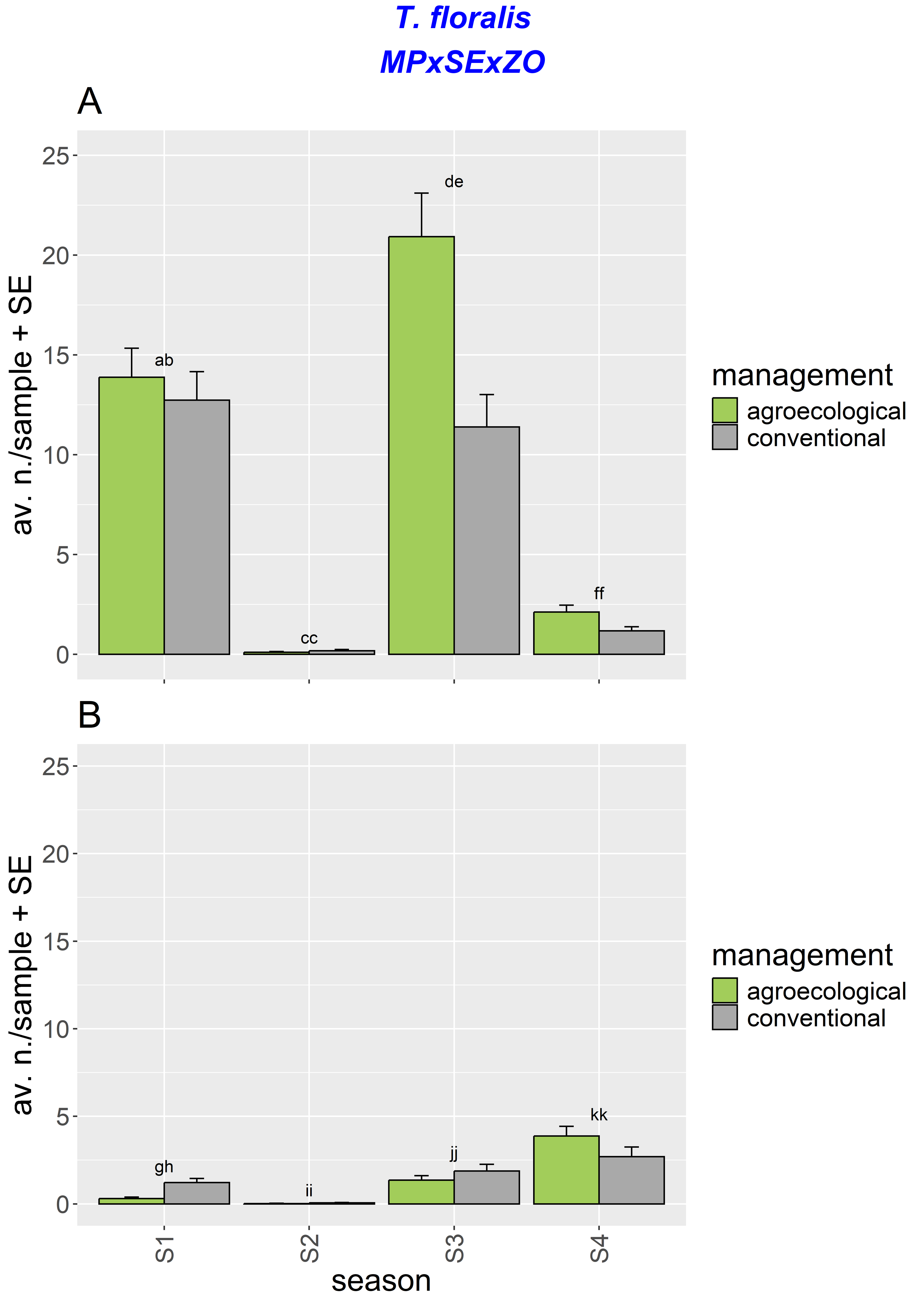

Supplement: S1 Fig — The bar plots illustrate the significant interaction of management x zone x season (MPxZOxSE) across (A) the plateau and (B) the mountainous. Significance letters for the pairwise tests are indicated (see Table 1). (TIF) [file pone.0327126.s007.tif]

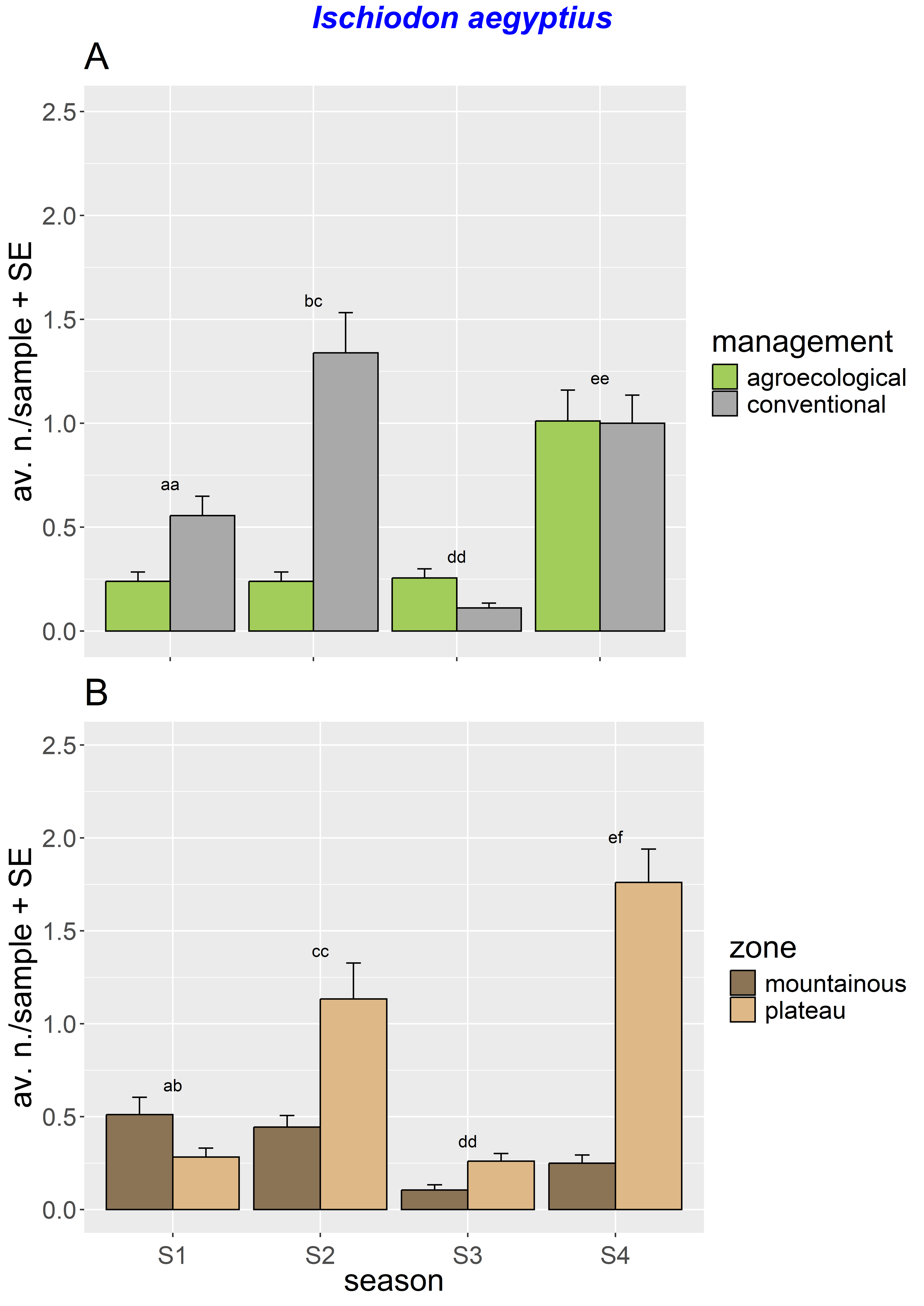

Supplement: S2 Fig — The bar plots illustrate the significant interactions of: (A) management x season (MPxSE) and (B) zone x season (ZOxSE). Significance letters for the pairwise tests are indicated (see Table 1). (TIF) [file pone.0327126.s008.tif]

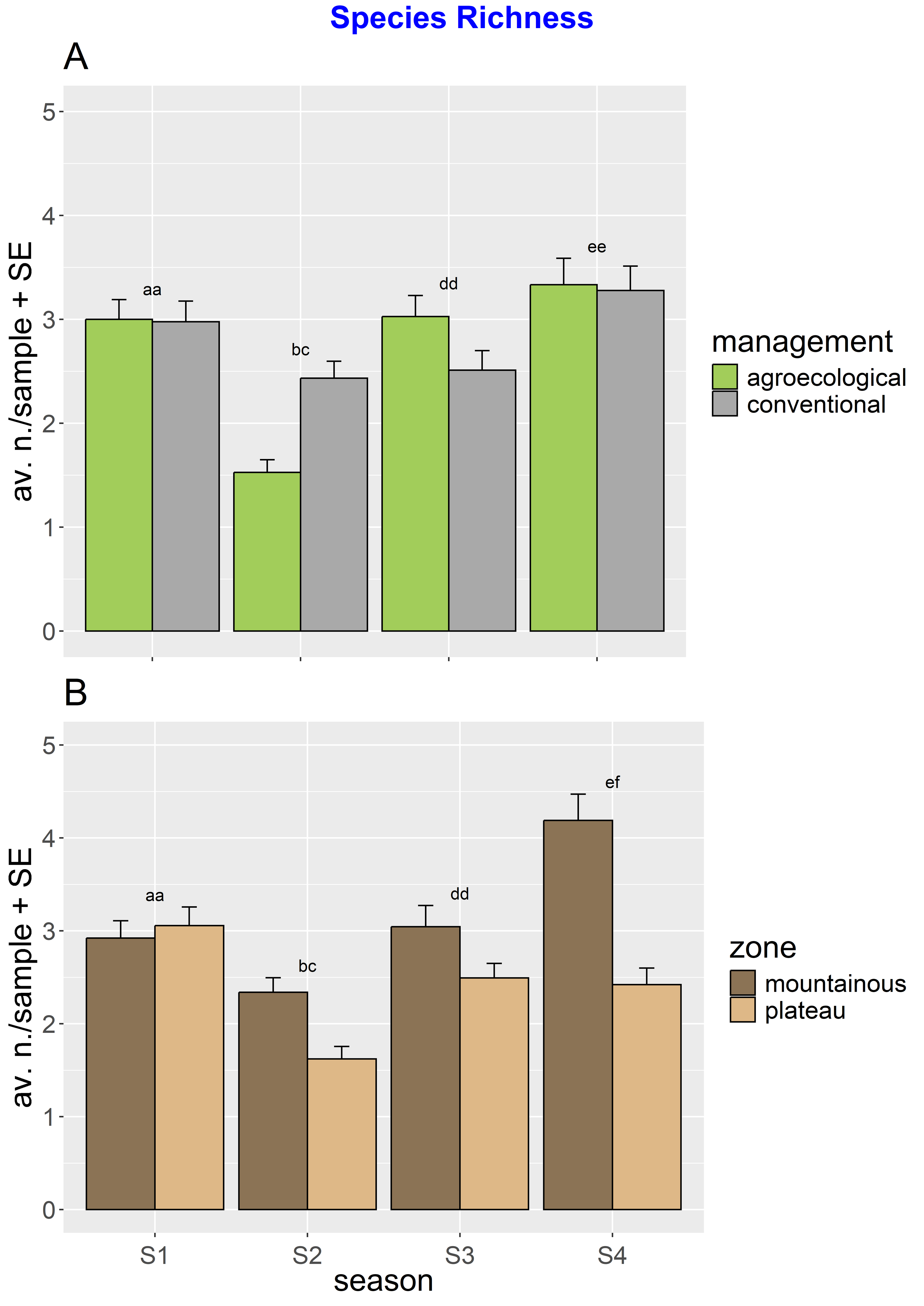

Supplement: S3 Fig — The bar plots illustrate the significant interactions of: (A) management x season (MPxSE) and (B) zone x season (ZOxSE). Significance letters for the pairwise tests are indicated (see Table 2). (TIF) [file pone.0327126.s009.tif]

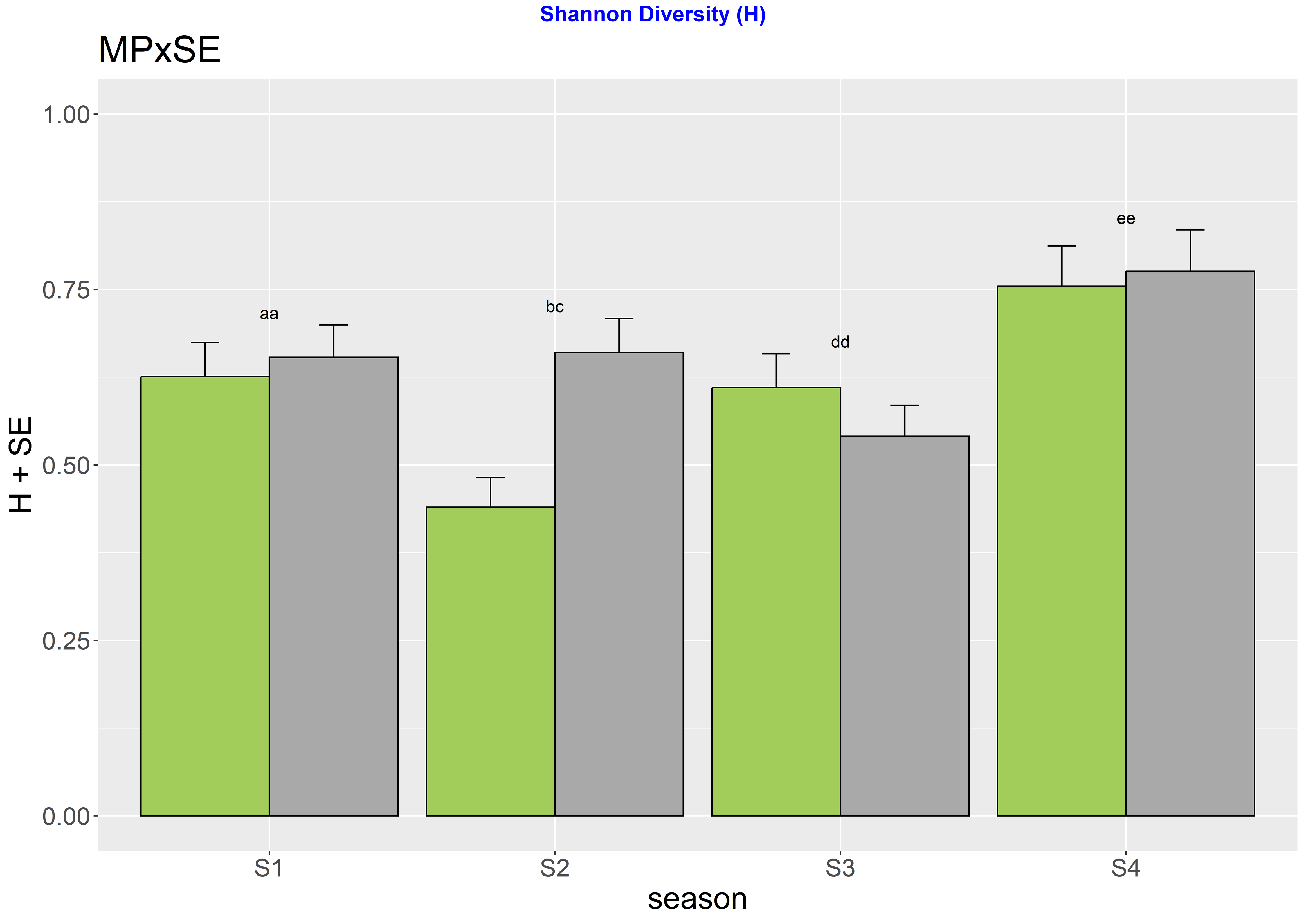

Supplement: S4 Fig — Shannon Diversity. The bar plots illustrate the significant interactions of management x season (MPxSE). Significance letters for the pairwise tests are indicated (see Table 2). (TIF) [file pone.0327126.s010.tif]

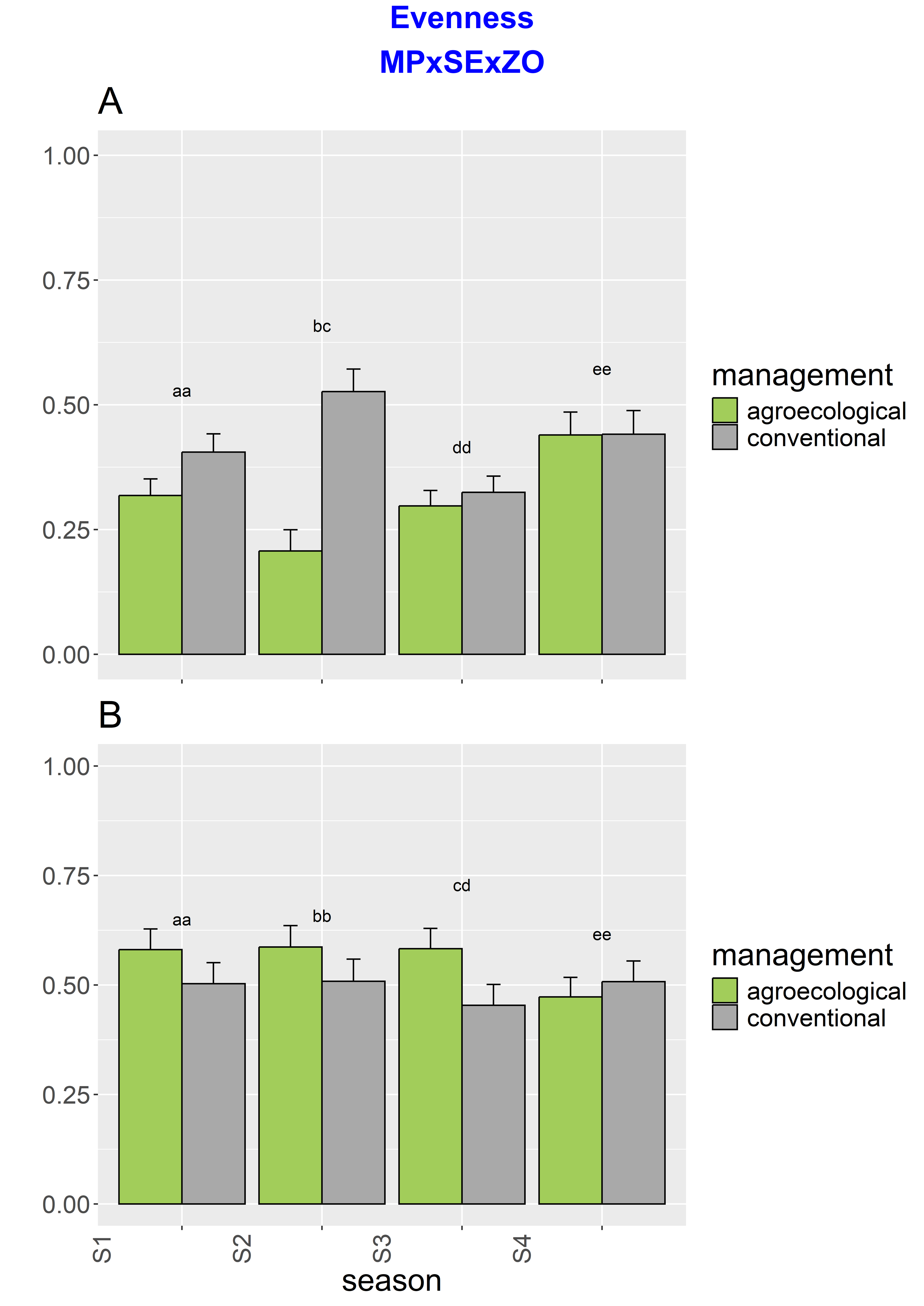

Supplement: S5 Fig — The bar plots illustrate the significant interactions of management x zone x season (MPxZOxSE) across (A) the plateau and (B) the mountainous. Significance letters for the pairwise tests are indicated (see Table). (TIF) [file pone.0327126.s011.tif]
